# Supplementary material for: Evaluation of a Non-Face-to-Face Multidisciplinary Health Care Model in a Population with Rheumatoid Arthritis Vulnerable to COVID-19 in a Health Emergency Situation
Source: Healthcare (Basel). 2021 Dec 17;9(12):1744. doi: 10.3390/healthcare9121744 (PMC8701032; doi:10.3390/healthcare9121744)
Supplement: Supplementary file 1 [file healthcare-09-01744-s001.zip › healthcare-1456931-supplementary.pdf]

Supplementary material

Table S1 Study dimensions and exploratory thematic questions

Study dimensions	Thematic script (patients seen in face-to-face appointments)	Thematic script (patients seen in telemedicine appointments)	Thematic script (professionals)
Therapeutic relationship	What was the communication with the professionals like? Mention the barriers or facilitators of face-to-face consultation with respect to communicating with the professionals.	What was the communication with the professionals like? Mention the barriers or facilitators of telemedicine with respect to communicating with the professionals.	What was the communication with the patients or their caregivers like? Mention the barriers or facilitators of telemedicine with respect to communicating with the patients or caregivers.
	What was the therapeutic relationship (trust, empathy, agreements) with the professionals who treated you like?	What was the therapeutic relationship (trust, empathy, agreements) with the professionals who treated you like?	What was the therapeutic relationship with your patients or their caregivers like?
Technological resources	What resources do you consider necessary to be treated by telemedicine at home?	What was involved in setting up a telemedicine appointment with the IPS?	What was involved in using ICT technological resources for treatment and evaluation?
		What technological resources (ICTs) did you have to enable a doctor visit by telemedicine? What other resources do you think would be important for implementing telemedicine?	What other resources do you think could optimize telemedicine?
		What was involved in using technological resources (ICTs) for telemedicine from your home?	Were the patient's needs met with the technological resources used?
Telemedicine experiences	As a patient with rheumatoid arthritis who is being treated by regular face-to-face appointments, what has your experience been like in the context of the SARS-COV-2/COVID-19 national contingency?	As a patient with rheumatoid arthritis who is being treated by regular telemedicine appointments, what has your experience been like in the context of the SARS-COV-2/COVID-19 national contingency?	What was involved in setting up a telemedicine appointment with the IPS? What was the impact of the above on the care and health outcomes of the patients you treated (adherence to therapy, complications, emergency room visits-hospitalization)?
Acceptability-feasibility of the	———	Do you think that the methodology and	Do you think that the methodology and

model		resources (time, materials...) in the telemedicine model were sufficient and adequate for their care/follow-up? Tell us why.	resources (technical difficulties, time, materials) in the telemedicine model were sufficient and appropriate to meet the objectives of the model? Tell us why.<br>What are the difficulties and obstacles to implementing a telemedicine model in the context of Bogota and the Colombian Health System?
Reasons for not accepting the telemedicine appointment (in the group that decided to see the doctor in person).	What were your reasons for not accepting telemedicine care?	———	———

Source: prepared by authors. ICTs: information and communication technologies. IPS: Health service provider institution

Table S2 Changes in medicine by group from model to model, and in the group as a whole

	Medicines used in the telemedicine model						
	Visit 1<br>n=71		Visit 2<br>n=71		Visit 3<br>n=71		*p*-value
	Frequency	(%)	Frequency	(%)	Frequency	(%)	
Analgesics							
Acetaminophen	49	69,0	24	33,8	41	57,7	0,345
Amitriptyline	0	0,0	0	0,0	0	0,0	
Codeine	2	2,8	4	5,6	3	4,2	1,000
Duloxetine	0	0,0	0	0,0	0	0,0	
Hydrocodone	8	11,3	6	8,5	3	4,2	0,337
Oxycodone	0	0,0	0	0,0	0	0,0	
Pregabalin	2	2,8	1	1,4	1	1,4	1,000
Tramadol	5	7,0	3	4,2	3	4,2	0,795
Trazodone	1	1,4	1	1,4	1	1,4	1,000
Antimalarials							
Chloroquine	6	8,5	6	8,5	5	7,0	0,938
Hydroxychloroquine	0	0,0	0	0,0	0	0,0	
b/ts DMARDs							
Abatacept	0	0,0	0	0,0	0	0,0	
Adalimumab	2	2,8	2	2,8	2	2,8	1,0
Certolizumab	6	8,5	6	8,5	6	8,5	1,0
Etanercept	10	14,1	9	12,7	10	14,1	1,0
Golimumab	5	7,0	5	7,0	5	7,0	1,0
Infliximab	0	0,0	1	1,4	1	1,4	1,0
Rituximab	1	1,4	0	0,0	0	0,0	1,0
Tocilizumab	1	1,4	1	1,4	1	1,4	1,0
Tofacitinib	3	4,2	4	5,6	4	5,6	1,0
csDMARDS							
Azathioprine	0	0,0	0	0,0	0	0,0	
Leflunomide	38	53,5	38	53,5	36	50,7	0,927

Methotrexate	40	56,3	39	54,9	35	49,3	0,673
Mycophenolate	0	0,0	0	0,0	0	0,0	
Sulfasalazine	9	12,7	9	12,7	7	9,9	0,834
GCs							
Betamethasone	5	7,0	4	5,6	1	1,4	0,353
Deflazacort	3	4,2	3	4,2	3	4,2	1,000
Methylprednisone	0	0,0	0	0,0	0	0,0	
Prednisone	40	56,3	40	56,3	33	46,5	0,397

Additional medicines in the whole group of patients

	Visit 1 n=218		Visit 2 n=201		Visit 3 n=206		p-value
	Frequency	(%)	Frequency	(%)	Frequency	(%)	
Analgesics							
Amitriptyline	1	0,5	0	0,0	1	0,5	1
Duloxetine	1	0,5	0	0,0	0	0,0	1
Pregabalin	6	2,8	6	3,0	4	1,9	0,815
Trazodone	12	5,5	11	5,5	9	4,4	0,837

Changes in medicines by group in the whole group of patients

	Visit 2 n=200		Visit 3 n=177		p-value
	Frequency	(%)	Frequency	(%)	
Analgesics					0,023
NP/WC	152	76	131	74	
Increase	17	8,5	8	4,5	

Decrease	9	4,5	5	2,8	
Start	3	1,5	15	8,5	
Discontinue	12	6	12	6,8	
Switching to another within the same class	7	3,5	6	3,4	
b/ts DMARDs					0,260
NP/WC	191	95,4	168	94,8	
Increase	0	0	1	0,6	
Decrease	0	0	0	0	
Start	3	1,5	6	3,4	
Discontinue	5	2,5	1	0,6	
Switching to another within the same class	1	0,6	1	0,6	
csDMARDs					0,234
NP/WC	161	80,5	147	83	
Increase	14	7	7	4	
Decrease	11	5,5	9	5,1	
Start	3	1,5	7	4	
Discontinue	10	5	4	2,2	
Switching to another within the same class	1	0,5	3	1,7	
GCs					0,889
NP/WC	174	87	151	85,3	
Increase	10	5	10	5,7	
Decrease	8	4	11	6,2	
Start	5	2,5	4	2,3	
Discontinue	2	1	1	0,6	
Switching to another within the same class	1	0,6	0	0	

NP/WC: non-prescribed /Without changes

Table S3 Changes in Drug Categories during follow up in the five sub-groups of patients analyzed

	**Telemedicine model**			**Face-to-face usual care model**			**Face-to-face Usual Care model > Telemedicine model> Telemedicine model**			**Telemedicine model > Telemedicine model> face-to-face Usual Care model**			**Face-to-face Usual Care model > Telemedicine model > face-to-face Usual Care model**		
	**Visit 2 n=71**	**visit 3 n=63**		**Visit 2 n=18**	**Visit 3 n=15**		**Visit 2 n= 48**	**Visit 3 n= 38**		**Visit 2 n= 17**	**Visit 3 n= 18**		**Visit 2 n= 23**	**Visit 3 n= 23**	
	**n (%)**	**n (%)**	**p value***	**n (%)**	**n (%)**	**p value***	**n (%)**	**n (%)**	**p value***	**n (%)**	**n (%)**	**p value***	**n (%)**	**n (%)**	**p value***
**Analgesics**			0.001			0.659			0.175			0.689			1.000
NP/WC	61 (85.9)	42 (66.6)		12 (66.7)	10(66.5)		33 (68.8)	28 (73.7)		13(76.5)	15 (83.3)		17 (73.8)	17 (73.8)	
Increase	5 (7.1)	3 (4.8)		3 (16.7)	1 (6.7)		4 (8.3)	1 (2.6)		1 (5.9)	2 (11.1)		2 (8.7)	1 (4.4)	
Decrease	2 (2.8)	0 (0)		2 (11.1)	1 (6.7)		2 (4.2)	2 (5.3)		1 (5.9)	1 (5.6)		1 (4.4)	1 (4.4)	
Start	0 (0)	8 (12.7)		0 (0)	1 (6.7)		2 (4.2)	5 (13.2)		0 (0)	0 (0)		0 (0)	1 (4.4)	

Discontinue	2 (2.8)	7 (11.1)		0 (0)	1 (6.7)		5 (10.4)	0 (0)		2 (11.8)	0 (0)		2 (8.7)	3 (13)	
Switching to another within the same class	1 (1.4)	3 (4.8)		1 (5.5)	1 (6.7)		2 (4.2)	2 (5.3)		0 (0)	0(0)		1 (4.4)	0 (0)	
**b/ts DMARDs**			1.000			1.000			0.195			0.514			0.739
NP/WC	68 (95.8)	62 (98.4)		18 (100)	15 (100)		44 (91.7)	35 (92.1)		17 (100)	17 (94.4)		21 (91.2)	20 (86.9)	
Increase	0 (0)	0(0)		0 (0)	0 (0)		0 (0)	0 (0)		0 (0)	1 (5.6)		0 (0)	0 (0)	
Decrease	0 (0)	0 (0)		0 (0)	0 (0)		0 (0)	0 (0)		0 (0)	0 (0)		0 (0)	0 (0)	
Start	2(2.8)	1(1.6)		0 (0)	0 (0)		1 (2.1)	3 (7.9)		0 (0)	0 (0)		0 (0)	2 (8.7)	
Discontinue	1(1.4)	0 (0)		0 (0)	0 (0)		3 (6.3)	0 (0)		0 (0)	0 (0)		1 (4.4)	1 (4.4)	
Switching to another within the same class	0 (0)	0 (0)		0 (0)	0 (0)		0 (0)	0 (0)		0 (0)	0 (0)		1 (4.4)	0 (0)	
**cs DMARDs**			0.093			0.685			0.125			1.000			1.000
NP/WC	67(94.4)	52(82.5)		9 (50)	11 (73.3)		36 (75)	30 (79)		14 (82.4)	15 (83.3)		20 (86.9)	20 (86.8)	
Increase	1 (1.4)	3 (4.8)		4 (22.2)	2 (13.3)		2 (4.2)	0 (0)		2 (11.8)	1 (5.6)		2 (8.7)	1 (4.4)	
Decrease	1 (1.4)	4 (6.3)		2 (11.1)	1 (6.7)		7 (14.6)	2 (5.3)		0 (0)	1 (5.6)		0 (0)	0 (0)	
Start	0 (0)	3 (4.8)		1 (5.6)	0 (0)		0 (0)	2 (5.3)		1 (5.9)	1 (5.6)		0 (0)	1 (4.4)	
Discontinue	1 (1.4)	0 (0)		2 (11.1)	1 (6.7)		3 (6.3)	2 (5.3)		0 (0)	0 (0)		1 (4.4)	1 (4.4)	

Switching to another within the same class	1 (1.4)	1 (1.6)		0 (0)	0 (0)		0 (0)	2 (5.3)		0 (0)	0 (0)		0 (0)	0 (0)	
**GCs**			0.705			0.698			0.755			0.229			0.577
NP/WC	69 (97.2)	59 (93.7)		14 (77.8)	11(73.3)		41 (85.4)	32 (84.2)		17 (100)	15 (83.3)		16 (69.6)	18 (78.3)	
Increase	1 (1.4)	1 (1.6)		1 (5.5)	2 (13.3)		1 (2)	2 (5.3)		0 (0)	3 (16.7)		3 (13.1)	0 (0)	
Decrease	1(1.4)	2 (3.1)		2 (1.1)	2 (13.3)		3 (6.3)	3 (7.9)		0 (0)	0 (0)		2 (8.7)	2 (8.7)	
Start	0 (0)	1 (1.6)		1 (5.6)	0 (0)		3 (6.3)	1 (2.6)		0 (0)	0 (0)		1 (4.4)	2 (8.7)	
Discontinue	0 (0)	0 (0)		0 (0)	0 (0)		0 (0)	0 (0)		0 (0)	0 (0)		1 (4.4)	1 (4.4)	
Switching to another within the same class	0 (0)	0 (0)		0 (0)	0 (0)		0 (0)	0(0)		0 (0)	0 (0)		0 (0)	0 (0)	

Switched drugs categories during follow up in the five groups of patients analyzed. *Statistical differences were determined to be significant at p-values less than 0.05. >: Transition between models; b/ts DMARDs: biological/targeted synthetic Disease-modifying antirheumatic drugs; csDMARDs: conventional synthetic Disease-modifying antirheumatic drugs; GCs: Glucocorticoids; NP/WC NP: non prescribed or WC: Without change. See description of the whole group at supplementary file 1: Table S1.

Table S4 Outcomes and RA treatment description of patients who were infected by SARS-Cov2.

>: Transition between models; AF: Atrial fibrillation; AH: Arterial Hypertension; bDMARDs: biological Disease-modifying antirheumatic drugs; COPD: Chronic Obstructive Pulmonary Disease; CQ: Chloroquine; CTG: Chronic tophaceous gout; ETN: Etanercept; GLM:

Case No.	Sex	Age	Duration of RA (years)	Consultation model	RA treatment	Comorbidities	Clinical course of SARS-Cov2 infection	Outcomes	Observation
1	F	66	23	TM	bDMARD (ETN) + Prednisone + MTX	AH - OA – AF	Hospitalized	Complete recovery	AH without treatment – AF treatment with rivaroxaban
2	F	81	1	UC>TM>UC	bDMARD (RTX) + Prednisone	AH – Osteoporosis – OA – Hypothyroidism			
3	F	66	45	UC	SSZ+Prednisone	AH			
4	F	59	16	UC>TM>TM	MTX+LFN+Prednisone+ bDMARD (GLM)	OA - Hypothyroidism – Epilepsy – Depressive disorder – LTBI treated.	At home	Complete recovery	Two months with positive sample. Two samples positive? Reinfection?
5	M	65	6	UC>TM>UC	MTX+LFN+Prednisone + Betamethasone (single dose IM)	AH – OA – EP – PCa – Colonic polyps	Asymptomatic	Complete recovery	Pre-operatory sample was positive. Before

Golimumab; IM: Intramuscular injection; LFN: Leflunomide; LTBI: Latent Tuberculosis infection; MTX: Methotrexate; OA:

									schedule surgery had positive test
6	M	68	29	UC>TM>TM	LFN+Prednisone	AH – Type 2 Diabetes Mellitus – OA – Osteoporosis - Chronic gastritis.		Complete recovery	
7	M	73	14 years	UC>TM	SSZ+Prednisone +CQ	AH – COPD- OA – Osteoporosis - CTG	Hospitalized seven days.	Died due to pulmonary involment	

Osteoarthritis; PCa: Prostate cancer; PE: Pulmonary embolism; RA: Rheumatoid Arthritis; RTX: Rituximab; SARS-Cov2: Severe acute respiratory syndrome coronavirus 2; SSZ: Sulfasalazine; TM: Telemedicine model; UC: Face-to-face usual care model.
